# Identification and functional characterisation of Complement Regulator Acquiring Surface Protein-1 of serum resistant *Borrelia garinii *OspA serotype 4

**DOI:** 10.1186/1471-2180-10-43

**Published:** 2010-02-10

**Authors:** Nathalie D van Burgel, Peter Kraiczy, Tim J Schuijt, Peter F Zipfel, Alje P van Dam

**Affiliations:** 1 Department of Medical Microbiology, Centre of Infectious Diseases, Leiden University Medical Centre, PO Box 9600, 2300 RC, Leiden, the Netherlands; 2 Institute of Medical Microbiology and Infection Control, University Hospital of Frankfurt, Paul-Ehrlich-Str. 40, D-60596 Frankfurt, Germany; 3 Department of Infection Biology, Leibniz-Institute for Natural Products Research, Beutenbergstr.11a, 07745 Jena, Germany

## Abstract

**Background:**

*B. burgdorferi *sensu lato (sl) is the etiological agent of Lyme borreliosis in humans. Spirochetes have adapted themselves to the human immune system in many distinct ways. One important immune escape mechanism for evading complement activation is the binding of complement regulators Factor H (CFH) or Factor H-like protein1 (FHL-1) to Complement Regulator-Acquiring Surface Proteins (CRASPs).

**Results:**

We demonstrate that *B. garinii *OspA serotype 4 (ST4) PBi resist complement-mediated killing by binding of FHL-1. To identify the primary ligands of FHL-1 four CspA orthologs from *B. garinii *ST4 PBi were cloned and tested for binding to human CFH and FHL-1. Orthologs BGA66 and BGA71 were found to be able to bind both complement regulators but with different intensities. In addition, all CspA orthologs were tested for binding to mammalian and avian CFH. Distinct orthologs were able to bind to CFH of different animal origins.

**Conclusions:**

*B. garinii *ST4 PBi is able to evade complement killing and it can bind FHL-1 to membrane expressed proteins. Recombinant proteins BGA66 can bind FHL-1 and human CFH, while BGA71 can bind only FHL-1. All recombinant CspA orthologs from *B. garinii *ST4 PBi can bind CFH from different animal origins. This partly explains the wide variety of animals that can be infected by *B. garinii*.

## Background

*Borrelia burgdorferi *sensu lato (sl), the etiologic agent of Lyme borreliosis, is a genetically diverse species. The different genospecies of *B. burgdorferi *sl appear to be associated with different manifestations of the disease [1,2]. *B. burgdorferi *sensu stricto (ss) is more common in North America but also found in Eurasia and is associated with arthritis, while *B. garinii *and *B. afzelii *are only present in Eurasia and are more commonly associated with Lyme neuroborreliosis and cutaneous manifestations, respectively. Specifically *B. garinii *OspA serotype 4 (ST4) strains, a genetically homogenous group, are frequently observed as a causative agent of neuroborreliosis in adults in Europe [3-6]. Recently it has also been proposed, though not yet generally accepted, to delineate the *B. garinii *ST4 strains as a separate species, *B. bavariensis*, due to large differences compared to *B. garinii *non-ST4 in multilocus sequence analysis (MLSA) on several housekeeping genes [7].

The different human pathogenic genospecies are associated with certain human serum resistance profiles; the majority of *B. burgdorferi *ss and *B. afzelii *strains are relatively resistant to human serum, while most *B. garinii *strains are highly sensitive to complement-mediated killing in vitro. Among *B. garinii*, the ST4 strains showed a similar resistant profile as *B. burgdorferi *ss and *B. afzelii *[8-10].

*B. burgdorferi *sl has developed a variety of immune evasion strategies, among which the binding of two host-derived fluid-phase regulators of complement: Factor H (CFH) and Factor H-like protein 1 (FHL-1). CFH and FHL-1 the main immune regulators of the alternative pathway of complement activation, are structurally related proteins composed of several protein domains termed short consensus repeats (SCRs) [11]. CFH is a 150-kDa glycoprotein composed of 20 SCR domains. In contrast, FHL-1 is a 42-kDa glycoprotein corresponding to a product of an alternatively spliced transcript of the *cfh *gene and consists of seven SCRs. The seven N-terminally located SCRs of both complement regulators are identical with the exception of four additional amino acids at the C-terminus of FHL-1 [12]. CFH and FHL-1 in the human host are responsible for preventing binding of factor B to C3b, supporting the dissociation of the C3bBb complex and acting as a cofactor for factor I-mediated cleavage of C3b, the central component of the three complement activation pathways [12-15].

Serum resistant *Borrelia *acquire CFH and/or FHL-1 by direct interaction with outer surface proteins designated CRASPs (Complement Regulator-Acquiring Surface Proteins) [16]. Previously, five different CRASPs have been described for *B. burgdorferi *ss and *B. afzelii*. The CFH and FHL-1 binding CspA protein is (also designated CRASP-1) encoded by *cspA*, a gene located on the lp54 plasmid. Although the lp54 plasmid of *B. burgdorferi *and *B. afzelii *carries multiple genes encoding a number of paralogous proteins, also called the gbb54 orthologous family, only the CspA is capable of binding human CFH and FHL-1 [17]. CspA is upregulated by spirochetes during the tick-mammalian transmission stage and down regulated during persistent infection [18,19]. CspZ is a distinct protein encoded by the *cspZ *gene located on plasmid lp28-3 and is expressed at higher levels during the mammalian infection than in bacteria residing in ticks or during laboratory cultivation [18]. Anti-CspZ antibodies can be detected as early as two weeks post infection in mice infected by ticks [20]. CspZ has been shown to bind other yet unknown proteins and therefore can have multiple functions [19-22]. The CFH-binding CRASP proteins BbCRASP-3, -4, and -5 belong to the OspE-related proteins (Erp) paralogous family and their respective genes are located on diverse cp32 prophage DNA molecules [23]. Erp proteins are expressed in tissues in the host during disseminated mammalian infection. Erp proteins have also been shown to be able to bind to factor H related proteins-1 (CFHR1) and plasminogen [24-29].

In contrast to *B. burgdorferi *ss and *B. afzelii *most *B. garinii *strains are unable to bind human complement regulators [30]. Two CspA orthologs from *B. garinii *ST6 ZQ1, named BgCRASP-1α and BgCRASP-1β, have been shown to bind weakly to FHL-1 but not to human CFH [31]. Little data is published on complement evasion strategies of human serum resistant strains of the *B. garinii *ST4 strains. The gbb54 orthologous family of *B. garinii *ST4 has not been studied before.

It has been elaborately shown which gbb54 ortholog from *B. burgdorferi *ss and *B. afzelii *can bind human CFH, but little is known about the function of the other orthologs. It has been described previously that CspA derived from *B. burgdorferi *ss interacts with human CFH; however none of the closely related protein of the gbb54 family, interacts with human CFH [32]. Wallich et al characterised all gbb54 orthologous members of a *B. afzelii *and *B. garinii *strain wherein none of the remaining orthologs could bind human CFH/FHL-1 [17,31]. We hypothesise that orthologs from the gbb54 family have the ability to bind to CFH from several animal origins.

The aim of the present study was to investigate the mechanism for complement evasion by *B. garinii *ST4 strains and to isolate and functionally characterize the specific gbb54 orthologs binding to human CFH/FHL-1 and also to other mammalian and avian CFH. We could prove binding of 2 ST4 gbb orthologs, BGA66 and BGA71, to human FHL-1, whereas BGA66 also bound CFH. Moreover, both these and other orthologs from the gbb54 family were also able to bind CFH from various animal species.

## Results

### Serum susceptibility testing of borrelial strains

**Figure 1:**
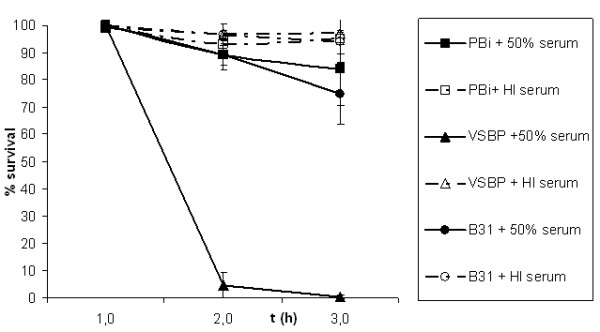
**In vitro serum susceptibility of *B. garinii *ST4 PBi, *B. garinii *non-ST4 VSBP, and *B. burgdorferi *ss B31**. Resistance to complement was determined by counting motile spirochetes by dark-field microscopy and values obtained were represented as percentages of survival. All strains were tested in triplicate with 50% NHS and HiNHS. VSBP is rapidly killed by complement, while >75%of *B. burgdorferi *ss B31 and *B. garinii *ST4 PBi are alive after 3 hours of incubation.

To assess and to compare serum susceptibility of *B. garinii *PBi and VSBP as well as *B. burgdorferi *ss B31, spirochetes were incubated for 3 h with either 50% NHS or 50% HI NHS. As shown in Fig 1, >75% of the cells of *B. garinii *ST4 PBi and *B. burgdorferi *ss B31 survived in serum, indicating that both strains resist complement-mediated killing. In contrast, *B. garinii *non-ST4 strain VSBP was highly sensitive to complement as 99% of the cells were immobilized and showed blebs after 3 hours. Incubation of strains PBi, VSBP, and B31 with HI NHS resulted in no or very little immobilisation. Summarising *B. garinii *ST4 PBi and *B. burgdorferi *ss B31 are resistant to human serum when incubated with active human complement, while *B. garinii *non-ST4 VSBP is not human serum resistant.

### The detection of the membrane attack complex deposited on borrelial cells after complement activation

**Figure 2:**
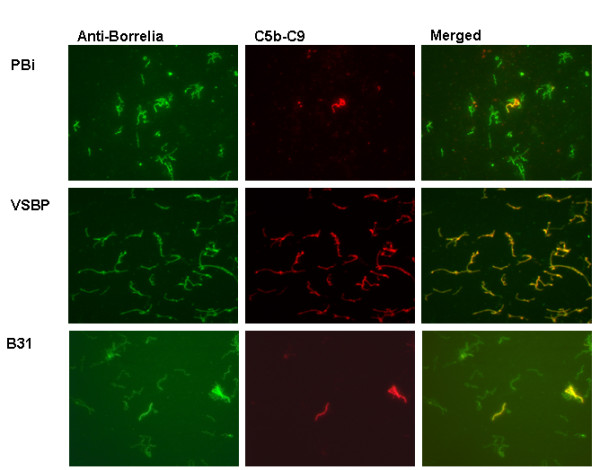
**Detection of deposited C5b-9 complex on the surface of *Borrelia *by Immunofluorescence microscopy**. *B. garinii *PBi and VSBP and *B. burgdorferi *ss B31 were incubated with 25% NHS and deposition of C5b-C9 was detected by a MAb. Few cells of *B. garinii *ST4 PBi stained positive for C5b-C9, while almost all spirochetes were covered with C5b-C9 using *B. garinii *non-ST4 VSBP. The absence of deposition of C5b-C9 onto *B. burgdorferi *ss B31 is comparable to *B. garinii *ST4 PBi.

To test whether membrane attack complex (MAC) was formed on the surface of different strains after complement activation, spirochetes were incubated with 25% serum and deposition of the MAC was detected by immuno-fluorescence microscopy (IF) (Fig 2). The majority of the cells of *B. garinii *ST4 PBi and *B. burgdorferi *ss B31 stained negative for the MAC while all *B. garinii *non-ST4 VSBP were fully covered with MAC. This finding indicates that *B. garinii *ST4 PBi and *B. burgdorferi *ss B31 allow formation of the MAC on their bacterial surface only to a limited extent in comparison to *B. garinii *non-ST4 strain VSBP.

### Detection of bound complement regulators to different borrelial strains

**Figure 3:**
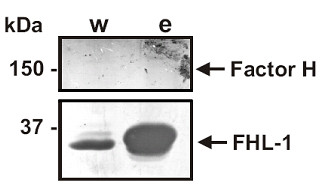
**Detection of bound complement regulators by *B. garinii *ST4 PBi**. After incubation of spirochetes with NHS-EDTA, bound proteins were eluted. The wash (w) and the eluate (e) fraction were separated by SDS-PAGE. The last wash and eluate fraction were subjected to SDS-PAGE and separated proteins were blotted on nitrocellulose. CFH and FHL-1 were visualised using a polyclonal goat anti-factor CFH antiserum (Calbiochem). It is shown that *B. garinii *ST4 PBi is able to bind FHL-1 on its membrane.

In order to elucidate the capability of serum resistant *B. garinii *ST4 PBi to bind complement regulators CFH and FHL-1 to the surfaces in a non-denatured state, intact spirochetes were incubated with NHS which was supplemented with EDTA to prevent complement activation. Complement regulators were allowed to adsorb to the *Borrelia *surface and bound proteins were subsequently eluted with acidified 0.1 M glycine. The wash and the eluate fraction were analyzed for the presence of CFH and FHL-1 by Western blotting. As shown in Fig 3, FHL-1, but not CFH could be detected in the eluate fraction indicating that *B. garinii *ST4 PBi specifically interact with FHL-1.

### Accessibility and surface exposure of CFH/FHL-1 binding proteins of *B. garinii *ST4 PBi

**Figure 4:**
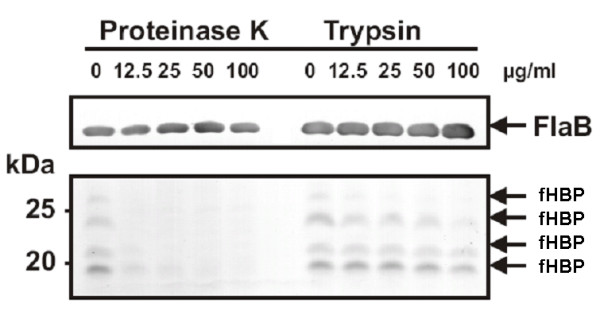
**Accessibility of CFH/FHL-1 binding proteins of *B. garinii *ST4 PBi by different proteases**. Spirochetes of *B. garinii *ST4 PBi were incubated with either proteinase K or trypsin at concentrations of 12.5 to 100 μg/ml or in buffer without any protease (0). After 1 h of incubation, cells were lysed by sonication as described in Materials and Methods. Each protein lysate was then subjected to 10% Tris/Tricine SDS-PAGE, blotted to membranes, and analyzed by Western or ligand affinity blotting. CFH/FHL-1 binding proteins were identified using NHS and a polyclonal anti-CFH antibody. Equal sample loading was assessed by detection of flagellin (FlaB) using MAb L41 1C11 1C11 at a dilution of 1:1000. Mobilities of molecular mass standards are indicated to the left. Four proteins able to bind CFH/FHL-1 and they are readily digested by proteinases and therefore located on the membrane.

In order to identify FHL-1 binding proteins produced by *B. garinii *ST4 PBi and to determine whether these proteins are exposed to the extracellular space, spirochetes were treated with increasing concentrations of proteinase K or trypsin and proteolysis was detected by ligand affinity blotting. Cell lysates obtained after protease treatment were separated by SDS-PAGE, transferred to nitrocellulose and the respective proteins were detected. As shown in Fig 4, four distinct binding proteins could be detected in untreated serum-resistant *B. garinii *ST4 PBi. Treatment with proteinase K at the lowest concentration resulted in the complete elimination of CFH/FHL-1 binding. Upon treatment with trypsin, degradation was only achieved at a concentration of 100 μg/μl. As expected, the intracellular protein flagellin was resistant to trypsin and proteinase K treatment, even at the highest concentration. These data demonstrate that *B. garinii *ST4 PBi produced up to four surface-exposed CFH/FHL-1 binding proteins, in the range of 19-26 kDa. This is in concordance to the findings of McDowell et al, where *B. garinii *ST4 PBi expressed a 20.5 and 26 kDa protein that were found to interact with CFH [33]. The CspA orthologs tested in this study are in the range of 25-27 kDa, the smaller proteins detected appear to belong to the Erp protein family.

### Cloning and identification of the CFH/FHL-1 binding proteins of *B. garinii *ST4 PBi

Assuming that the genes encoding CFH/FHL-1 binding proteins of *B. garinii *ST4 PBi share similarity to CspA encoding *cspA *gene of *B. burgdorferi *ss B31, *B. afzelii *MMS and *B. garinii *ZQ1, a database search was conducted. Four genes revealed a high degree of similarity with either CspA of *B. burgdorferi *ss B31, *B. afzelii *MMS or *B. garinii *ZQ1 as described previously [31,34]. BGA66, BGA67, BGA68 and BGA71 showed similarity to previously described CspA of about 50%. Comparative sequence analysis, revealed that orthologs BGA66 and BGA71 were found to have the highest degree of similarity within the putative CFH/FHL-1 binding regions of CspA (region 1-3)[35-37]. BGA66, BGA67, BGA68 and BGA 71 as well as CspA of *B. burgdorferi *ss strain B31 were cloned and expressed as GST fusion proteins.

### Determination of binding of CspA orthologs to CFH and FHL-1

**Figure 5:**
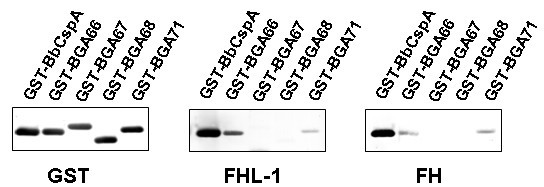
**Binding capabilities of CFH and FHL-1 to CspA orthologs of *B. garinii *ST4**. Purified GST fusion proteins, BbCspA, BGA66, BGA67, BGA69, and BGA71 (500 ng/lane) were subjected to 10% Tris/Tricine SDS-PAGE and blotted to nitrocellulose membranes. Membranes were then incubated with recombinant FHL-1 or with NHS. GST-fusion proteins were detected by using anti-goat GST antibody and binding to CFH and FHL-1 were visualized using mAb VIG8 specific for the C-terminal region of CFH and αSCR1-4 antiserum specific for the N-terminal region of FHL-1. Binding of CFH and FHL-1 is visible for BGA66 and BGA71.

Binding of CFH and FHL-1 to non-denatured purified recombinant proteins was evaluated by ligand affinity blot. Proteins were separated under denaturing conditions and subsequently blotted on a nitrocellulose membrane. As shown in Fig 5, BbCspA used as positive control bound strongly to CFH and FHL-1 as described previously [34]. Orthologs BGA66 and BGA71 were capable of binding to both complement regulators, however, with reduced intensities compared to CspA.

**Figure 6:**
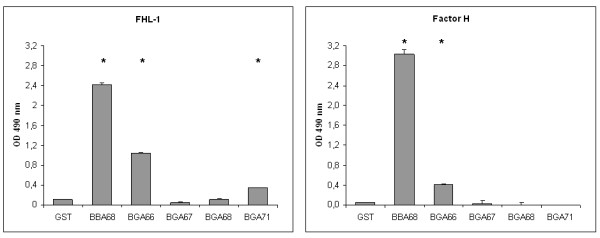
**Binding of CspA orthologs to FHL-1 and CFH**. Recombinant proteins (500 ng each) were coated onto an ELISA plate and incubated with purified FHL-1 (A) and CFH (B). Binding was assayed by ELISA using polyclonal αSCR1-4 that recognized CFH and FHL-1. All experiments were performed at least in triplicate. * (p < 0.05 compared to baseline (GST) OD)

To further confirm binding of CspA orthologs an ELISA was conducted. CspA orthologs BGA66, BGA67, BGA68, and BGA71 were immobilized on a microtiter plate and binding of CFH and FHL-1 was evaluated (Fig 6). BbCRASP-1 used as a positive control strongly bound to CFH and FHL-1. Of the four CspA orthologs analyzed, BGA66 was capable of binding to both complement regulators, this binding was significantly higher than the baseline (p < 0.05). Ortholog BGA71 specifically bound to FHL-1 (p < 0.05) but less efficiently than CspA and BGA66. In contrast, orthologs BGA67 and BGA68 were not able to bind to CFH or FHL-1 at all.

These data confirmed that orthologs BGA66 as well as BGA71 derived from *B. garinii *ST4 PBi were capable of binding FHL-1. Binding of CFH in both assays is evident for BGA66, but not for BGA71.

### Mapping of the binding domains of CFH and FHL-1 to CspA orthologs

**Figure 7:**
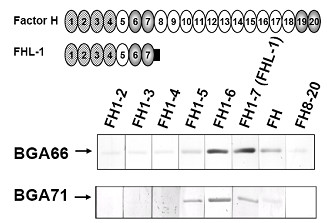
**Mapping of the binding domains of CFH and FHL-1 for BGA66 and BGA71**. Schematic representation of the CFH and FHL-1 protein and ligand affininty blot analysis of fusion proteins. The complement regulatory domains SCR 1-4 are in checked. Purified recombinant protein was separated by 10% Tris-Tricine-SDS-PAGE and transferred to nitrocellulose. Membranes were incubated with either recombinant FHL-1 (FH1-7) or several deletion constructs of CFH (FH1-2, FH1-3, FH1-4, FH1-5, FH1-6, FH8-20) or with human serum (FH). Bound proteins were visualized using polyclonal goat anti-CFH (Calbiochem), or MAb VIG8 directed against the C-terminus of CFH. SCR 5-7 are essential SCR for binding of BGA66 and BGA71 to interact with CFH/FHL-1.

In order to map the binding regions of CFH and FHL-1 interacting with BGA66 and BGA71, various deletion constructs of CFH and FHL-1 were used for ligand affinity assays (Fig 7). BGA66 bound to full-length CFH and FHL-1, but to none of the deletion constructs lacking SCRs 5-7. BGA71 bound FHL-1 as well as deletion constructs SCR1-5 and SCR1-6. Thus, SCR5-7 of both CFH and FHL-1 are required for binding to BGA66 and BGA71.

### Expression of BGA66 and BGA71 by real-time RT-PCR

cDNA prepared from in vitro cultured *B. garinii *ST4 PBi were tested in a quantitative real time PCR. Cultures repeated in sexplet demonstrated a mean expression of BGA66 of 34 copies/1000 copies *flaB *(SD 22) and BGA71 21 copies/1000 copies *flaB *(SD 18). All spirochetes cultivated in vitro expressed BGA66 and BGA71 simultaneously.

### Analysis of CFH binding of different animal sera to CspA orthologs

**Figure 8:**
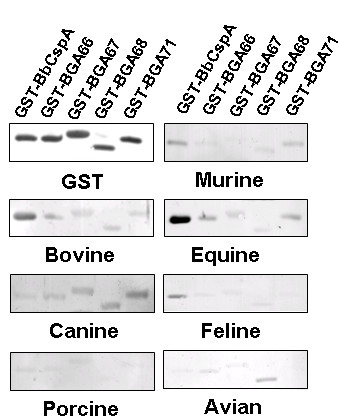
**Interaction of different CspA orthologs to interact with animal CFH**. Purified recombinant CspA and *B. garinii *ST4 CspA orthologs were subjected to 10% Tris/Tricine SDS-PAGE and blotted to nitrocellulose membranes. Recombinant proteins were visualized by an anti-GST antibody. Additional membranes were incubated with sera obtained from diverse animals. Interacting proteins were then visualized using a polyclonal anti-CFH antibody.

A variety of sera obtained from different animals were used to analyse binding of CFH to CspA, BGA66, BGA67, BGA68, and BGA71 by ligand affinity blotting. As shown in Fig 8, CspA orthologs displayed distinct capacity of binding to CFH from a wide variety of sera from different mammals and poultry. All orthologs exhibit binding of CFH from bovine, equine and canine serum with different intensities. BGA68 and BGA71 showed a weak binding capacity to murine CFH. In addition, BGA68 but not CspA nor other orthologs bound to avian CFH. Porcine and feline serum proteins did not bind any of the CspA orthologs of *B. garinii *ST4 PBi while feline CFH appears to bind only to BbCspA.

## Discussion

We are the first to demonstrate that *B. garinii *ST4 PBi is serum resistant and is able to acquire FHL-1 but not CFH from human serum. In addition, we identified two distinct CspA orthologs, BGA66 and BGA71 as potential ligands of complement regulators CFH and FHL-1. These proteins were produced under in vitro conditions as demonstrated by real time PCR. Finally, we demonstrated distinct binding capacities of CFH of different mammalian and avian origin to different CspA orthologs of serum resistant *B. garinii *ST4 PBi.

In Europe four human pathogenic genospecies are endemic. *B. burgdorferi *ss, *B. afzelii*, and *B. spielmanii *display a human serum resistant phenotype while *B. garinii *strains are often serum sensitive [8-10,38,39]. Within the OspA typing scheme, *B. garinii *ST4 strains represent a distinct branch as shown by random amplified polymorphic DNA (RAPD) analysis. On the basis of MLSA analysis it has recently been proposed, though not yet generally accepted, to delineate this subgroup in a separate species; *B. bavariensis *[7,40]. *B. garinii *ST4 is remarkably often associated with dissemination to the CNS [3,5,6,41]. In a previous study it was confirmed that *B. garinii *non-ST4 strains, including strains isolated from CSF, are sensitive to complement while *B. garinii *ST4 strains were resistant to human complement [10]. In this report we confirm with an in vitro killing assay and IF that *B. garinii *ST4 is resistant to human complement killing and that it does not allow formation of MAC on the spirochetal membrane.

It has been extensively shown that CspA fulfils a key role in complement resistance of *B. burgdorferi *ss [42,43]. In the present study, a comparative binding analysis was conducted to isolate and characterize CspA orthologs from the serum resistant, *B. garinii *ST4 strain PBi. We hypothesised that binding of CFH and/or FHL-1 via CspA orthologs contributes to serum resistance of *B. garinii *ST4 PBi. We identified orthologs BGA66 and BGA71 but not BGA67 and BGA68 as being potential ligands for FHL-1 and CFH. In vitro cultured spirochetes bound FHL-1 but not CFH on their surface. The affinity for FHL-1 appeared to be stronger than for CFH, it can be concluded that FHL-1 competes with CFH for the same binding site and thus CFH could not be detected in the cell binding assay. When employing ELISA on recombinant proteins, BGA66 bound both complement regulators while BGA71 only bound FHL-1. By ligand affinity blotting BGA71 bound FHL-1 as well as CFH. A logical explanation for this phenomenon might be the denaturing conditions of the Western blot, suggesting that native BGA71 specifically interacts with FHL-1 only.

Furthermore, it was previously shown that CspA forms homodimers and three regions of CspA have been implicated in formation of a functional binding site of CspA to CFH/FHL-1 [35-37,43]. Previously it has been hypothesised that the C-terminal YKXXDXXXP motif is important in binding of CFH and FHL-1, as well as the lysine residue at position 246 of CspA [31]. Recently it was also shown that a leucine residue at position 146 within the proposed CFH binding region 1 as well as Tyr240, Asp242 and Leu246 within the proposed binding region 3 of CspA were important in binding of CFH and FHL-1 [35]. The C-terminus of all known human CFH/FHL-1 binding CspA and the *B. garinii *ST4 gbb54 orthologs is shown in table 1. Comparative sequence analysis revealed that the C-terminus of BGA66 and BGA71 are highly homologous to the C-terminus of all known human CFH/FHL-1 binding CspA. Ortholog BGA66 contains the C-terminal motif as well as the Leu246, while BGA71 contains the C-terminal motif but has a phenylalanine instead of a leucine residue at position 246. Positions 146 and 240 are unchanged in BGA66 and BGA71 both orthologs show substitutions at position 242; the Asp242 in BGA66 and BGA71 is replaced by a glutamic acid and a threonine residue, respectively. A substitution of Asp242 by a neutral alanine residue within CspA did not have a significant effect on binding, while the replacement of aspartic acid by tyrosine at this position influenced binding of FHL-1 and is associated with a loss of binding of CFH [35]. Lack of binding of native BGA71 to CFH is likely to be due to the non-synonymous mutation of aspartic acid by threonine, while BGA66 can still bind both CFH and FHL-1 due to the synonymous mutation of aspartic acid to glutamic acid. It is likely that absence of CFH binding by BGA71 might be a result of an effect of the mutation on protein folding and conformation. Our finding that under denaturing conditions BGA71 can bind CFH, but not under native folded conditions supports this hypothesis.

**Table 1:** C-terminus of all CspA and *B. garinii *ST4 CspA orthologs

Protein		240										250	
BbCspA	Y	**Y**	**K**	*D*	F	**D**	T	*L*	K	P	A	F	Y
BaCspA	N	**Y**	**K**	*D*	L	**D**	S	*F*	N	P	I	N	-
BgCspAα	N	**Y**	**K**	*E*	F	**D**	P	*L*	N	L	D	Y	-
BgCspAβ	N	**Y**	**K**	*T*	L	**D**	S	*F*	K	S	I	N	-
BGA66	N	**Y**	**K**	*E*	H	**D**	S	*L*	K	P	I	Y	-
BGA67	N	**Y**	**K**	*E*	F	N	S	*L*	K	P	I	Y	-
BGA68	N	**Y**	**K**	*N*	L	H	S	*F*	K	T	V	Y	Y
BGA71	N	**Y**	**K**	*T*	L	**D**	S	*F*	K	P	I	N	-

C-terminal end of CspA orthologs described in this study and previously determined. Positions 242 and 246 depicted in italic. The sequence for CspA derived from *B. burgdorferi *ss B31, BaCspA from *B. afzelii *MMS, ZQA68 (BgCspAα) and ZQA71 (BgCspA β) from *B. garinii *ZQ1, BGA66, BGA67, BGA68 and BGA71 from *B. garinii *ST4 PBi.

A number of Gram-negative as well as Gram-positive bacteria have already been shown to be able to bind CFH in order to protect themselves from complement-mediated lysis [44-46]. CFH possess three binding sites for complement C3b, however the only essential binding site is SCR1-4 [12,47,48]. Here we show that BGA66 as well as BGA71 bind SCR5-7 of CFH and FHL-1, thus leaving the N-terminus free for maintaining their regulatory activity in factor I-mediated inactivation of C3b [34]. Our finding indicates that *B. garinii *ST4 strains can bind functionally active CFH and FHL-1 on the membrane by BGA66 and BGA71 in order to evade complement activation.

*B. burgdorferi *sl has developed an intriguing system to respond to changes of the microenvironments by coordinated expression of proteins. In vitro experiments usually do not completely mirror the expression patterns of CspA during the tick to mammal infectious cycle and might also vary in cultured population [49]. CspA shows a distinct expression profile as it is mainly expressed during transmission of spirochetes from the tick-to-mammal and mammal-to-tick infection cycle [19]. Previously antibodies to CspA could be detected in sera from infected mice and from Lyme disease patients suggesting prolonged expression of CspA in the mammalian host [50-52]. In the present study we demonstrated that in vitro *B. garinii *ST4 PBi is capable of expressing BGA66 and BGA71. Experiments regarding expression of BGA66 and BGA71 during tick-to-mammal transmission and mammalian infection are ongoing and will give more insight in their function in vivo.

Although all five CRASPs of *B. burgdorferi *sl are primarily identified as ligands of human complement regulators, several studies clearly showed that CspA can also bind CFH from other mammalian hosts [22]. CFH binding of several animal CFH sources has also been reported in a recent article where new CFH binding proteins were identified [53]. It is still not quite clear how the wide variety of complement resistance is obtained in strains that do not interact with human CFH. The *B. burgdorferi *ss and *B. afzelii *orthologs of CspA were previously not studied for binding to CFH of non-human origin. In this study all CspA orthologs of *B. garinii *ST4 PBi were tested with whole sera from different animals. BGA67 and BGA68 lack binding to human CFH but were able to interact with CFH from other hosts, of which some are not competent reservoir hosts for *Borrelia*. It is likely that several members of the gbb54 paralogous family are designated to bind CFH from other species in the infectious cycle and are therefore not redundant but essential for infection of a wide range of hosts. The interaction of mammalian CFH with CspA orthologs of *B. burgdorferi *sl might unveil a part of the serum resistance patterns obtained from in vitro experiments.

## Conclusions

In this study we demonstrated *B. garinii *ST4 PBi is able to evade complement killing and it can bind FHL-1 to membrane expressed proteins. Recombinant proteins BGA66 can bind FHL-1 and human CFH, while BGA71 can bind only FHL-1. All recombinant CspA orthologs from PBi can bind CFH from different animal origins. This can partly explain the wide variety of animals that *B. garinii *can infect.

## Methods

### Borrelial strains and culture conditions

*B. garinii *strains PBi and VSBP as well as *B. burgdorferi *ss strain B31 were cultured until mid-log phase (5 × 10^7 ^cells per ml) at 33°C in modified Barbour-Stoenner-Kelly (BSK-H) medium (Sigma). Aliquots of 1 ml were then diluted 1:1 with glycerol peptone (8% glycerol, 1% w/v Proteose Peptone 3 (Brunschwig chemie, Amsterdam) in distilled water), dispensed into screw-cap tubes (Nunc, Wiesbaden, Germany), frozen at -80°C, and used as stock cultures. Prior to use, a frozen suspension of spirochetes was thawed and inoculated into fresh BSK-H medium.

### Serum bactericidal assay

Serum susceptibility of *Borrelia *was determined as described previously [10]. Briefly, serum obtained from a non-immune human donor (NHS) was frozen at -80°C and thawed on ice prior to use. Heat inactivated (HI) serum was incubated for 1 hour at 56°C in order to inactivate complement. *B. garinii *ST4 PBi, *B. garinii *non-ST4 VSBP, and *B. burgdorferi *ss B31 were cultured until mid-log phase in BSK-H. An aliquot of 50 μl containing 10^7 ^live *Borrelia*/ml was added to 50 μl of serum and incubated for 1 and 3 h at 33°C. After incubation aliquots of 5 μl were drawn from the suspensions and mobility and blebbing of the spirochetes was assessed under dark-field microscopy. One hundred spirochetes were examined, motile cells as well as non-motile cells were counted and the percentage of survival was calculated. The experiment was repeated three times.

### Immunofluorescence assay

Immunofluorescence microscopy was performed as described previously [54].

Briefly, freshly cultured *B. garinii *strains PBi, VSBP, and *B. burgdorferi *ss B31 were incubated for 30 minutes in BSK-H medium containing 25% NHS. Subsequently spirochetes were washed twice with PBS/1% BSA, resuspended in the same buffer and air dried on microscope slides overnight. After fixation in 100% methanol, slides were incubated with human immune serum containing anti-*Borrelia *antibodies (1:2000) and a mAb recognizing a neoepitope of the terminal C5b-9 complex (1:1000) (DAKO). Slides were washed with PBS-1% BSA and incubated with an anti-human immunoglobulin G-fluorescein isothiocyanate-labeled antibody (1:100) (bioMérieux) and an anti-mouse immunoglobulin G Cy3-labeled antibody (1:1000) (Jackson). Afterwards slides were washed three times and mounted with Mowiol (Hoechst). Spirochetes were visualized by confocal microscopy using an Axioscop 2 mot plus fluorescence microscope (Carl Zeiss).

### Serum adsorption experiments

*Borrelia *(2 × 10^9 ^cells) were grown to mid-log phase, harvested by centrifugation (5,000 × *g*, 30 min, 4°C), and resuspended in 100 μl of veronal-buffered saline (supplemented with 1 mM Mg^2+^-0.15 mM Ca^2+^-0.1% gelatine, pH 7.4). To inhibit complement activation, NHS was incubated with 0.34 mM EDTA for 15 min at room temperature. The spirochete suspension was then incubated in 1.5 ml of NHS-EDTA for 1 hour at room temperature with gentle agitation. After three washes with phosphate-buffered saline (PBS) (supplemented with.15 M NaCl, 0.03 M phosphate, 0.02% sodium azide, pH 7.2), 0.05% Tween 20. The proteins bound to the cells were eluted by incubation with 0.1 M glycine-HCl, pH 2.0, for 15 min. Cells were removed by centrifugation at 14,000 × *g *for 20 min at 4°C, and supernatants were then analysed by Western blotting.

### Protease degradation assay

To characterize protease-susceptibility of CFH and FHL-1 binding proteins of *B. garinii *ST4 PBi, cells were treated with two different proteases as described previously [34]. Briefly, spirochetes were grown to mid-log phase, sedimented by centrifugation at 5,000 × g for 30 min, washed twice with cold PBS containing 5 mM MgCl_2 _(PBS-Mg), and resuspended in 100 μl PBS-Mg. To the *Borrelia *cell suspension (at a concentration of 10^8 ^in a final volume of 0.5 ml), proteinase K or trypsin was added to a final concentration of 12,5 μg/ml to 100 μg/ml. Following incubation for 1 hour at room temperature, proteolytic degradation with proteinase K or trypsin was terminated by the addition of 5 μl of phenylmethylsulfonyl fluoride or by the addition of 5 μl of phenylmethylsulfonyl fluoride and 5 μl of 4-(2-aminoethyl)-benzenesulfonyl fluoride, respectively. *Borrelia *were then gently washed twice with PBS-Mg, resuspended in 20 μl PBS-Mg, and lysed by sonication five times using a Branson B-12 sonifier (Heinemann, Schwäbisch Gmünd, Germany). Equal volumes of *Borrelia *lysates were subjected to Tris/Tricine SDS-PAGE, and proteins were transferred to nitrocellulose membranes as described previously [16]. Susceptibility of proteins to proteolytic degradation was assessed by Western or ligand affinity blotting with the appropriate monoclonal or polyclonal antibodies, followed by incubation with a horseradish peroxidase-conjugated IgG antibody, and then visualized by the addition of 3, 3', 5, 5'-tetramethylbenzidine.

### PCR cloning, expression and purification of recombinant CspA orthologous proteins

Sequences of genes encoding for CspA B31 and orthologs from *B. garinii *ST4 PBi were obtained from genbank (NC_006129 and NC_001857). Primers were designed using primer3 (MIT) and listed in table 2. Amplification reactions were performed in a 50 μl final volume, containing 25 μl IQ Supermix (Bio-Rad, Veenendaal, The Netherlands), 15 pmol forward primer, 15 pmol reverse primer, and 10 μl of a DNA isolate of cultured B31 or PBi. Following an enzyme activation step for 3 min at 95°C, amplification comprised 50 cycles of 30 s at 95°C, 30 s at 55°C and 30 s at 72°C. Genes lacking their leader sequences were ligated in frame into the pGEX-5X3 vector (Amersham Bioscience, Freiburg, Germany). The ligation mixtures were used to transform *Escherichia coli *MC1061. Plasmid DNA was prepared from the presumptive clones with the QIAprep kit (QIAGEN, Hilden, Germany), and the *Borrelia *DNA inserts were sequenced using the BigDye Terminator cycle sequencing kit (Applied Biosystems International, Foster City, CA, USA) in accordance with the manufacturers' recommendations. Plasmids were used to transform *E. coli *BL21. Expression of the GST fusion proteins was done by induction of the respective BL21 clones induced for 5 hours with 1 mM IPTG, followed by affinity purification with glutathione-Sepharose 4B (GE Healthcare, Netherlands). Expression and purity of generated GST fusion proteins were confirmed by employing SDS-PAGE, and protein concentrations were determined by a Bradford assay (Bio-Rad, Munich, Germany).

**Table 2:** Oligonucleotides used in this study

Oligonucleotides	Sequence (5'-3')	Target
BBA68s	ATGCGGCCGTGTTGTGTTTTAGTTTGGAT	BBA68
BBA68as	GTGGGATCCCATGCGCACCTTTTAGCAA	BBA68
BGA66s	ATGCGGCCGTGTTTTTAGTTTGGGCTCT	BGA66
BGA66as	GTGGGATCCCATGTGCCGTTAATAAAAATTG	BGA66
BGA67s	ATGCGGCCGATCAAGTGCAACGTATTTTT	BGA67
BGA67as	GTGGGATCCCATGTGCCGTTAATAAAAATTG	BGA67
BGA68s	ATGCGGCCGACATTATTGTTTTTAGTTTGGACTCT	BGA68
BGA68as	GTGGGATCCCATGTGCTGATAAAACC	BGA68
BGA71s	ATGCGGCCCATTGTTGTTTTTGGTTTAGACTC	BGA71
BGA71as	GTGGGATCCCATGTGTGCTGTTGATAAAATAG	BGA71
qFlaBs	GCTTCTGATGATGCTGCTG	FlaB
qFlaBas	TCGTCTGTAAGTTGCTCTATTTC	FlaB
qFlaB Taqmanprobe	GAATTRGCAGTAACGG-FAM	FlaB
qBGA66s	AGTTGTGCAGCAGCAATTTT	BGA66
qBGA66as	ATCCAGATCCTTTAAAGAC	BGA66
qBGA71s	TTCATATAGGTTGCTAATGCG	BGA71
qBGA71as	TTGCACACTCAAAACCAAAAA	BGA71

### Real Time-PCR analysis

For determining expression in vitro cultures of PBi spirochetes grown to mid log phase were isolated. Nucleic acid was extracted with a QiaAmp *Mini Blood DNA *kit (Qiagen, Hilden, Germany).

Total nucleic acid was treated with DNAse and 1 μg RNA was reverse transcribed using iScript (Bio-Rad) according to the manufacturer's protocol. Primers and probe for the *flaB *gene were designed from an interspecies conserved region of *flaB *using the Beacondesigner and listed in table 2. Amplification reactions were performed in a 50-μl final volume, containing 25 μl IQ Supermix (Bio-Rad, Veenendaal, The Netherlands), 15 pmol forward primer, 15 pmol reverse primer, 2.5 mM MgCl_2_, 0.3 μM FlaB-probe, or 1 × Sybergreen (Molecular Probes), and 10 μl cDNA. Following an enzyme activation step for 3 min at 95°C, amplification comprised 50 cycles of 30 sec at 95°C, 30 s at 55°C and 30 s at 72°C in an iCycler IQ real-time detection system (Bio-Rad). The FlaB assay was optimized using a TA vector into which the complete *flaB *encoding gene from *B. burgdorferi *ss B31 had been cloned and had an analytical sensitivity of 1 copy per PCR in 0.9% saline.

Quantitative DNA analysis was performed using the Icycler IQ5 PCR system.

The relative starting copy number was determined by cycle threshold detection using Icycler relative quantification software (Roche).

### SDS-PAGE, ligand affinity blot analysis, and Western blotting

Purified recombinant fusion proteins (500 ng) were subjected to 10% Tris/Tricine-SDS-PAGE under reducing conditions and transferred to nitrocellulose as previously described [16,55]. Briefly, after transfer of proteins onto nitrocellulose, nonspecific binding sides were blocked using 5% (w/v) dried milk in TBS (50 mM Tris-HCl (pH 7.5), 200 mM NaCl,0.1% Tween 20 for 1 hour at room temperature. Subsequently, membranes were rinsed four times in TBS and incubated for 1 hour at room temperature with TBS containing recombinant FHL-1, pooled non-immune human serum (NHS), or non-immune animal sera. To detect the fusion proteins a goat anti-GST antibody (dilution 1:2,000) (GE Healthcare, Germany) was used. Polyclonal rabbit anti-SCR1-4 antiserum (αSCR1-4) (dilution 1:1,000) used for the detection of FHL-1 and monoclonal antibody (mAb) VIG8 (undiluted) against the C-terminus of CFH, are described elsewhere [15,56]. After four washings with 50 mM Tris-HCl (pH 7.5)-150 mM NaCl-0.2% Tween 20 (TBST), membranes were incubated for 1 hour with either a polyclonal rabbit antiserum recognizing the N-terminal region of CFH (αSCR1-4) or mAb VIG8, directed against the C-terminus of CFH. Following four washes with TBST, strips were incubated with a peroxidase-conjugated anti-rabbit IgG antibody or with a peroxidase-conjugated anti-mouse IgG antibody (DAKO, Glostrup, Denmark) for 1 hour at room temperature. Detection of bound antibodies was performed by using 3, 3', 5, 5'-tetramethylbenzidine as substrate.

### ELISA

Recombinant proteins (500 ng/well) were immobilized on wells of a microtiter plate overnight at 4ºC. Unspecific binding sites were blocked with 0.1% gelatin in PBS for 6 h at 4ºC. CFH (Calbiochem), or recombinant FHL-1 was added to the wells and left overnight at 4ºC. Polyclonal goat anti-CFH antibody (Calbiochem) was added for 3 h at room temperature, protein complexes were identified using secondary horseradish peroxidase-coupled antiserum. The reaction was developed with 1,2-phenylenediamine dihydrochloride (Dako-Cytomation), and absorbence was measured at 490 nm.

### Binding domains of CFH and FHL-1 to CspA orthologs

To identify domains of CFH and FHL-1 responsible for binding of BGA66 and BGA71, 500 ng purified recombinant protein was separated by 10% Tris/Tricine SDS-PAGE and transferred to nitrocellulose. Membranes were then incubated with either recombinant FHL-1 (FH1-7), deletion constructs of CFH (FH1-2, FH1-3, FH1-4, FH1-5, FH1-6, FH8-20, FH19-20), or human serum as source for CFH. Bound proteins were visualized using polyclonal goat anti-CFH antibody (Calbiochem), or mAb VIG8.

### Statistical analysis

All statistical analyses were done using SPSS 16.0 and Microsoft Excel software. The two-tailed Student t-test was used to analyze ELISA results. Values of p < 0.05 were considered to be significant.

## Abbreviations

BSK-H: modified Barbour-Stoenner-Kelly medium; CFH: factor H; FHL-1: Factor H like; CRASP: Complement Regulator Acquiring Surface Protein; HI: heat inactivated; IF: Immunofluorescence; mAB: monoclonal antibody; NHS; non-immune human serum; SCR: short consensus repeats; SDS-PAGE; sodium dodecyl sulfate - polyacrylamide gel electrophoresis; ST4: OspA serotype 4; PBS: phosphate-buffered saline

## Authors' contributions

NDvB and APvD conceived of the study. NDvB performed serum killing assays, PCR cloning and performed ligand affinity blots and ELISA and drafted the manuscript. PK supervised protein assays and performed cell binding assays and protease assay and edited the manuscript. TJS performed IF experiments. PFZ was responsible for all recombinant CFH and FHL-1 protein assays. APvD supervised the work and edited the manuscript. All authors read and approved the final manuscript.
